# Blood Lead Levels in Adults and Children of East Otago Communities, New Zealand: Findings From a Public Health Response to Lead Detected in Drinking Water

**DOI:** 10.1002/puh2.70273

**Published:** 2026-05-14

**Authors:** Melyssa Roy, Susan Jack, Callum Thirkell, Giles Graham, Andrew Anglemyer, Michael Butchard

**Affiliations:** ^1^ National Public Health Service—Southern, Health New Zealand, Te Whatu Ora Wakari Hospital Dunedin New Zealand; ^2^ Department of Medicine University of Otago Dunedin New Zealand; ^3^ National Public Health Service, Health New Zealand, Te Whatu Ora Wakari Hospital Dunedin New Zealand; ^4^ The New Zealand Institute for Public Health and Forensic Science New Zealand; ^5^ National Centre for Epidemiology and Population Health Australian National University Canberra Australia; ^6^ Department of Preventive and Social Medicine University of Otago Dunedin New Zealand

**Keywords:** blood lead levels, drinking water, lead, public health response

## Abstract

**Background:**

In 2020–2021, intermittent elevated lead levels were detected in drinking water from communities of East Otago, New Zealand (NZ). A public health response was initiated with blood lead level (BLL) testing offered to all residents to identify any individuals with elevated levels and to determine if lead‐contaminated water caused elevated BLLs in this population.

**Study Design:**

Cross‐sectional study.

**Methods:**

BLL testing was offered to all residents to investigate possible lead exposure. Residents also completed a risk factor survey.

**Results:**

The majority of adults and children in the affected area had a BLL test; those with a venepuncture BLL and completed survey (1057 adults and 152 children) were included in the final estimates. The geometric mean (GM) BLL of adults was 1.54 µg/dL (95% confidence interval [CI] 1.49–1.60); children (0–17 years) had a GM BLL of 1.11 µg/dL (95% CI 1.01–1.22). In adults, BLLs increased with age, and children had higher BLLs than young adults. Twenty‐nine adults and two children had BLLs exceeding the NZ notifiable threshold of 5 µg/dL.

**Conclusions:**

In adults and children aged over 5 years, BLLs were not consistently significantly different from prior national estimates measured in 2014–2016, suggesting that lead detected in the drinking water did not result in elevated BLLs in the exposed population. BLLs in these NZ children and adults were higher than reported in comparable high‐income countries, likely due to exposure from known sources such as old paint, remnants of leaded petrol and tapware. NZ requires ongoing public health action to reduce lead exposure.

## Graphical abstract

Blood lead levels (BLLs) in East Otago adults and children were similar to prior New Zealand estimates, with no consistent evidence of elevation in the exposed population. BLLs were higher than comparable high‐income countries, likely reflecting exposure from known sources. Ongoing public health action is required to reduce lead exposure.

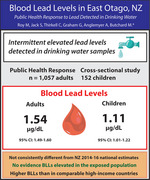

## 1 Introduction

Lead is a toxic heavy metal which has no biological function in humans [1]. Industrial activities and the use of leaded petrol and lead‐based paints have resulted in environmental pollution; consequently, all people have had some exposure to lead. Lead is a bioaccumulative toxin, which causes adverse health effects [2, 3]. Children are most vulnerable; irreversible neurocognitive and behavioural impairments may occur with lead poisoning at blood lead levels (BLLs) even below 5 µg/dL [4, 5]. Lead crosses the placenta and adversely affects the neurological development of unborn babies [6]. Chronic lead toxicity contributes to many diseases in both children and adults, including respiratory, neurological, gastrointestinal and cardiovascular illnesses [3, 7]. It is now recognised that there is no safe blood level of lead in children; even low levels may cause harm [8].

Public health action to reduce population exposure to lead has included removing lead from petrol, paint and food containers and regulating maximum levels in water, food and workplaces [5]. In countries with robust plumbing and drinking‐water regulation and monitoring, waterborne lead exposure is relatively uncommon compared to other pathways, such as exposure to lead paint and contaminated soil. Lead in drinking water may still occur due to corrosion of pipes and plumbing fixtures that contain lead. Drinking‐water legislation regulates the use of lead in plumbing components in many countries, but older pipes and plumbing fixtures that contain lead remain a risk [9].

### 1.1 Lead in New Zealand (NZ)

In NZ, lead‐based paint remained in use until 1978, so it is still present on many older houses. Undertaking home renovations poses a recognised risk for lead exposure due to direct contact and inhalation of paint flakes. Leaded petrol was phased out more slowly than many other high‐income countries, eventually being banned in 1996 [10]. Biomonitoring studies indicate that in the 1980s, NZ children had mean BLLs of 10.99 µg/dL [11]; however, like most other high‐income countries, mean BLLs have been decreasing since this time [5, 12]. The most recent national survey of BLLs in NZ was undertaken in 2014–2016, and this reported mean BLLs of 0.86 µg/dL in children (aged 5–18 years) and 1.31 µg/dL in adults [5]. Lead poisoning is notifiable in NZ, although routine screening is not undertaken. In 2021, NZ was in the process of updating the national guidelines on the management of lead toxicity with a reduction of the notifiable BLL from 10 to 5 µg/dL. Most lead poisoning occurs as a result of occupational exposures [13] or from exposure to lead‐based paint or shooting ranges [14]. The supply of safe drinking water in NZ is legislated; in 2020/21, 96% of the population received drinking water that complied with the national drinking‐water standards [15]. However, these standards do not require routine testing for lead in drinking water, and under the current national Building Code and standards [16], small amounts of lead are still permitted in plumbing fixtures in NZ.

### 1.2 Detection of Lead in Drinking Wate

In 2020, local government in Otago, NZ, initiated additional water testing in the East Otago townships of Waikouaiti, Karitane and Hawksbury Village to inform a corrosion monitoring strategy as part of a planned water treatment plant upgrade. Prior to this event, the drinking water supply had not been tested for lead for more than 20 years, so previous lead levels were unknown. Over a period of 6 months, intermittent elevated lead levels in the drinking water were detected from sampling taps in the distribution network. Exceptionally high lead levels that were 20–100 times higher than the maximum allowable value (MAV) of 0.01 mg/L were detected at peripheral drinking‐water distribution sites in December 2020. In January 2021, a lead level above the MAV was recorded in the raw water reservoir of the townships’ drinking water. As a precautionary approach, a Do Not Drink Notice was issued by the city council, as it was unclear what was causing the elevated readings and whether the community was at risk.

Two paths of action were undertaken. The first was a full investigation by the local government to identify the cause of the elevated lead levels detected at distribution sample taps and in the raw water. During the investigation, alternative water sources were provided to residents. The second course of action was a public health response, initiated by the local public health service, supported by the local primary health organisation and the Ministry of Health. Community BLL screening, together with a survey to ascertain risk factors, was offered immediately to all residents in the affected area.

The purpose of offering BLL testing was for individuals to be informed of their own BLLs and risk factors for lead exposure and to determine if the BLLs of the people in the affected communities were higher than expected. This strategy was to evaluate if any significant exposure to lead through the drinking water had occurred and to address the high level of community concern about possible health risks.

Two community meetings were held to provide information about lead toxicity, to describe the drinking‐water findings and to offer community BLL testing. A second meeting was held to present the findings from the BLL testing and provide an expert panel that included a paediatrician, toxicologist and environmental scientists to answer further questions from members of the public. Information about the health risks of lead was made available online and provided in printed versions. District public health officials spoke with national and regional media sources throughout the event to provide relevant and up‐to‐date information to the public.

### 1.3 Objectives of the Public Health Response

- To prevent any further possible exposure to lead from drinking water.
- To offer BLL testing to all residents in the affected area.
- To identify any individuals with elevated BLLs above the threshold of 5 µg/dL in order to offer appropriate risk assessment and management.
- To provide information and reassurance to the community.

### 1.4 Objectives of the Analysis of BLLs and Survey Data

- To compare the BLLs in the population of the affected area with the most recent national surveys of BLLs undertaken elsewhere in NZ, in order to explore whether the elevated lead levels in the drinking water affected BLLs in residents of Waikouaiti, Karitane and Hawksbury.

## 2 Methods

### 2.1 Data Collection

A BLL test was offered to all residents in the affected areas (Waikouaiti, Karitane and Hawksbury). A community pop‐up clinic provided free testing for a 1‐week period in February 2021, or residents could request a BLL test by attending their general practice (GP). Younger children were offered less invasive screening by heel‐prick capillary testing instead of venepuncture, at the discretion of the parents and phlebotomist. Capillary screening was utilised to improve testing uptake—venous phlebotomy can be a significant barrier to testing in young children. The heel‐prick test has high sensitivity for detecting clinically concerning BLLs when appropriate cutoff values are used [17]. Any screening results above the threshold of 5 µg/dL detected were confirmed with follow‐up venepuncture; this was a conservative threshold to ensure no children with elevated levels were missed.

Those who had a BLL test also completed either a paper or electronic self‐reported lead exposure risk assessment survey (Appendix 1). The survey captured information on demographics, water source, school, occupation, previous lead exposure, diet and other potential risk factors. The survey included written consent for de‐identified collation, analysis and reporting of results.

### 2.2 Inclusion and Exclusion Criteria

All residents in the area supplied with drinking water from the Waikouaiti, Karitane and Hawksbury water supply scheme were eligible for BLL testing as part of the public health response. A resident was defined as having lived in the area for at least 1 month in the last 12 months or for 2 weeks within the last 12 months for pregnant women and children—this reduced timeframe was chosen due to their greater vulnerability to lead exposure and higher potential absorption. East Otago contains many holiday homes, so the shorter timeframes were set in order to include people who may have been exposed but are not permanent residents. The timeframes were determined based on advice from a clinical toxicologist to capture the exposure duration at which measurable health effects were possible, though not necessarily likely, given the maximum measured drinking‐water lead levels.

Inclusion criteria for the analysis were (a) to be a resident in the described area, (b) to have a recent confirmed laboratory venous BLL and (c) to have provided a consented survey response. Adults and children of all ages were included.

### 2.3 Statistical Analysis

The analyses were stratified by age: as children (less than 18 years old) and adults (18 years or older). To assess the extent of coverage, the study population was compared to the 2018 census population estimates of residents in the area, and descriptive analyses by age, sex and ethnicity (Māori [indigenous ethnic group in NZ] and non‐Māori) were also performed.

BLLs were log‐transformed prior to analysis to satisfy linear regression assumptions and reduce the influence of extreme values. The geometric mean (GM) and 95% confidence intervals (CIs) were calculated and reported for all participants who received venepuncture tests. Individual BLLs were compared to both the existing notifiable threshold of 10 µg/dL and the newly proposed notifiable BLL threshold of 5 µg/dL.

Comparisons were made between the results from the current analysis and the most recently published population‐based estimates of BLL in NZ.

Multivariable regression models were built using presumed risk factor variables. Covariates were identified using a Wald test with a *p* value cut‐off point of *p* < 0.20. A backward elimination approach was employed to remove independent variables from the initial multivariable model that were both non‐significant (*α* = 0.05) and determined not to be a confounding variable based on their effect on other model parameters. All models were adjusted for clustering within households. Cases with missing survey responses were excluded from analyses. Analyses were performed in R.

## 3 Results

**FIGURE 1:**
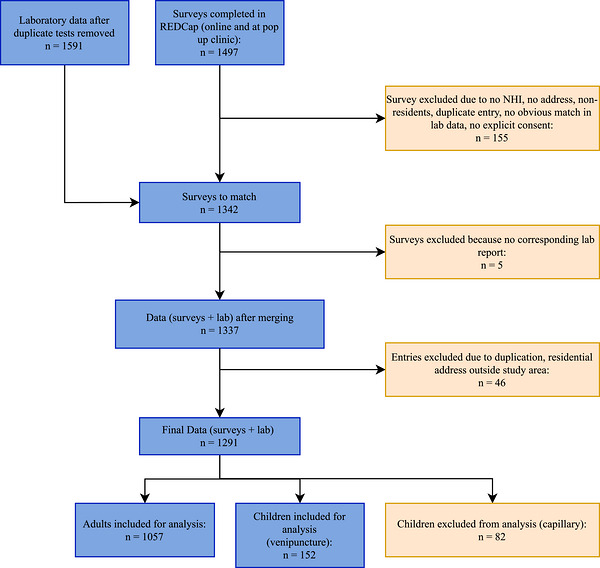
Flow of participants from testing to analysis.

Community testing clinics were very well attended, with most residents in the area attending to have their BLLs tested. A smaller proportion acquired their blood tests through their local GP. Most people also completed the matched questionnaire (*n* = 1498). After exclusions for non‐residents, duplicates and unmatched surveys, 1057 adults were included in the final analysis. Out of the 234 children tested, 152 had venepuncture BLLs and were also included in the final analysis (Figure 1).

On the basis of the most recent 2018 census data, most residents had BLL testing undertaken; approximately 95% of children were screened by capillary test or by venepuncture, and 71% of the adult population had a venous BLL measured. The study population was broadly reflective of the census estimates.

### 3.1 Blood Lead Levels

The GM of BLL of all participants in this study was 1.48 µg/dL (95% CI 1.43–1.53), with a higher BLL for adults (18 years or older) than for children (0–17 years) (1.54 µg/dL; 95% CI 1.49–1.60 and 1.11 µg/dL; 95% CI 1.01–1.22, respectively). Males (all age groups) had a higher BLL (1.69 µg/dL; 95% CI 1.61–1.79) than females (1.34; 95% CI 1.29–1.40). Non‐Māori (all age groups) had a higher BLL (1.51 µg/dL; 95% CI 1.46–1.56) than Māori (1.22 µg/dL; 95% CI 1.07–1.39) (Table 1).

**TABLE 1:** Distribution of the study population by select demographics and the respective blood lead levels (µg/dL) in East Otago communities in 2021.

	Total		Males		Females	
Age group (years)	*n* (%)^a^	GM (95% CI)	*n* (%)^a^	GM (95% CI)	*n* (%)^a^	GM (95% CI)
All	1209 (100)	1.48 (1.43–1.53)	501 (100)	1.69 (1.61–1.79)	708 (100)	1.34 (1.29–1.40)
0–17	152 (12.6)	1.11 (1.01–1.22)	88 (17.6)	1.22 (1.07–1.38)	64 (9.0)	0.98 (0.86–1.12)
18+	1057 (87.4)	1.54 (1.49–1.60)	413 (82.4)	1.82 (1.72–1.92)	644 (91.0)	1.39 (1.32–1.45)
0–4	26 (2.2)	1.82 (1.36–2.44)	16 (3.2)	1.92 (1.28–2.87)	10 (1.4)	1.67 (1.02–2.75)
5–9	53 (4.4)	1.06 (0.92–1.22)	34 (6.8)	1.04 (0.85–1.26)	19 (2.7)	1.10 (0.90–1.36)
10–17	73 (6.0)	0.97 (0.86–1.08)	38 (7.6)	1.16 (1.00–1.36)	35 (5.0)	0.79 (0.68–0.92)
18–19	10 (<1.0)	0.63 (0.50–0.81)	3 (<1.0)	0.60 (0.25–1.43)	7 (<1.0)	0.65 (0.47–0.90)
20–29	73 (6.0)	0.95 (0.82–1.10)	33 (6.6)	1.20 (0.94–1.53)	40 (5.7)	0.78 (0.66–0.92)
30–39	129 (10.7)	1.13 (1.03–1.23)	42 (8.4)	1.41 (1.22–1.63)	87 (12.3)	1.01 (0.90–1.13)
40–49	140 (11.6)	1.26 (1.15–1.39)	54 (10.8)	1.63 (1.40–1.90)	86 (12.2)	1.08 (0.96–1.20)
50–59	173 (14.3)	1.58 (1.45–1.73)	62 (12.4)	1.84 (1.58–2.14)	111 (15.7)	1.46 (1.31–1.62)
60–65	149 (12.3)	1.71 (1.56–1.87)	60 (12.0)	1.87 (1.62–2.15)	89 (12.6)	1.61 (1.44–1.80)
66+	383 (31.7)	1.96 (1.87–2.07)	159 (31.7)	2.21 (2.05–2.39)	224 (31.7)	1.80 (1.68–1.93)
Māori^b^						
All	108 (100)	1.22 (1.07–1.39)	50 (100)	1.41 (1.13–1.75)	58 (100)	1.08 (0.93–1.25)
0–17	36 (33.3)	1.07 (0.86–1.33)	21 (42.0)	1.18 (0.84–1.66)	15 (25.9)	0.93 (0.72–1.20)
18+	72 (66.7)	1.30 (1.11–1.53)	29 (58.0)	1.60 (1.19–2.15)	43 (74.1)	1.13 (0.94–1.36)
Non–Māori^b^						
All	1083 (100)	1.51 (1.46–1.56)	446 (100)	1.74 (1.65–1.83)	637 (100)	1.37 (1.30–1.43)
0–17	114 (10.5)	1.12 (1.01–1.25)	66 (14.8)	1.24 (1.08–1.42)	48 (7.5)	0.98 (0.83–1.56)
18+	969 (89.5)	1.56 (1.50–1.62)	380 (85.2)	1.84 (1.74–1.95)	589 (92.5)	1.40 (1.34–1.47)

Abbreviations: CI, confidence interval; GM, geometric mean.

^a^ Includes only those tested by venepuncture.

^b^ *n* = 18 unknown ethnicities excluded.

**FIGURE 2:**
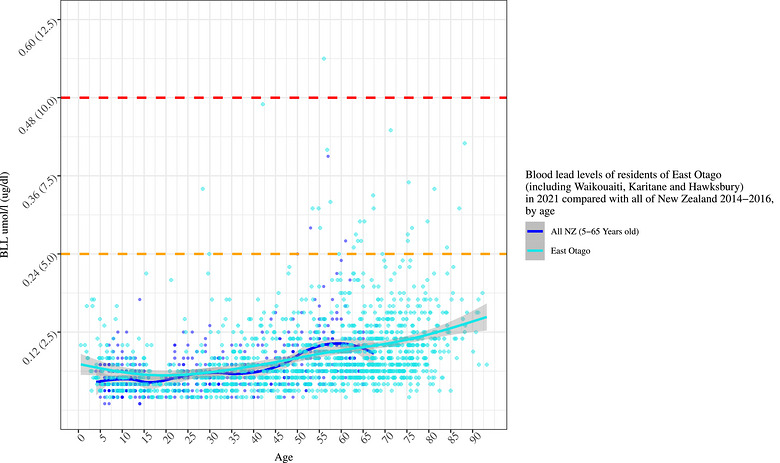
Blood lead levels of residents of East Otago (including Waikouaiti, Karitane and Hawksbury) in 2021 compared with all of New Zealand (2014–2016), by age. BLL, blood lead level; NZ, New Zealand.

The BLLs varied with age; BLLs increased with each age group from age 20 years (Table 1, Figure 2). Accordingly, in adults, the highest BLL was among the oldest age group (66 years or older) (GM = 1.96 µg/dL; 95% CI 1.87–2.07). Children in all age groups had higher BLLs than young adults aged under 30 years, with the highest BLLs in children detected in those aged under 5 years (GM = 1.82 µg/dL; 95% CI 1.36–2.44) (Table 1, Figure 2). Multivariable analysis showed predictors of BLLS in Table 2.

**TABLE 2:** Predictors of blood lead levels in adult males, females and children in East Otago, New Zealand.

Characteristics	EC	95% CI
Adult males		
Age (years)	1.01	1.01–1.02
Ship building	1.67	1.04–2.68
High‐risk job	1.34	1.12–1.62
Auto work	1.51	1.09–2.10
Adult females		
Age (years)	1.02	1.01–1.02
Shellfish	1.17	1.07–1.27
High‐risk job	1.30	1.00–1.68
Children (aged 0–17 years)		
Age (years)	0.95	0.93–0.97
House with peeling paint/recent renovations	1.52	1.10–2.11
House year		
After 1965	Ref.	—
1945–1965	1.15	0.92–1.42
Pre‐1945	1.42	1.11–1.81

Abbreviations: CI, confidence interval; EC, exponentiated coefficient of multivariable model; Ref., reference group.

### 3.2 BLLs Detected Above Notifiable Threshold

Of the 1057 adults tested, one had a BLL that exceeded the current notifiable level (10 µg/dL). An additional 28 adults had a BLL that met or exceeded the newly proposed notifiable level of 5 µg/dL. Amongst the 29 adults with BLLs at or above the 5 µg/dL threshold, most (26/29, 90%) were aged 55 years old or older, and 48% (14/29) were over 65 years of age.

Six children in total returned a high capillary BLL; they were subsequently retested in the subsequent weeks by venepuncture. Two of these children were found to have BLLs above the notifiable level (5 µg/dL); one had a BLL that also exceeded the current notifiable level of 10 µg/dL (data points not shown). Out of all 152 children tested by venepuncture, only these two children had elevated BLLs above threshold.

The six children who were retested due to elevated capillary BLLs were removed in a sensitivity analysis to examine for selection bias resulting from specifically including children with presumed high BLL in the population‐level BLL estimates. Children with screening capillary BLLs below the 5 µg/dL threshold were not retested or included in the analysis.

Follow‐up investigations by health protection officers identified potential exposures to known sources of lead (such as home renovations and occupational exposure) for most individuals under 60 years old with levels above the threshold.

## 4 Discussion

We have reported a public health response to intermittent elevated levels of lead detected in samples of drinking water in the townships in East Otago, NZ. The public health response was timely, precautionary and appropriate to evaluate the risk posed to the population [18].

The local government's final report found that the most likely cause of the elevated lead levels in the network was leaching from pipes and fittings in customer connections, and the most likely cause of the elevated lead level in the raw water was lead sediment, due to a recent spike in turbidity or capture of particulate material from the sample tap [19].

The public health response resulted in good uptake of the BLL testing. Reassuringly, few people were found to have BLLs above the notifiable threshold, and for most of these individuals, known risk factors were identified. These results indicate that significant long‐term exposure to lead from the water supply was unlikely.

### 4.1 Comparison of BLLs to Prior NZ Estimates

Over the last two decades, there has only been a single study examining NZ population BLLs, undertaken in 2014–2016 [5], so data for comparison are limited. However, most adults and children in this study had BLLs that did not differ significantly from prior estimates, suggesting that additional exposure from drinking water was unlikely.

#### 4.1.1 Adults

In this study, the GM for BLL in adults 18–65 years was 1.34 µg/dL (95% CI 1.28–1.41) (data not shown), which was not significantly different from the NZ study estimate of 1.31 µg/dL (95% CI 1.23–1.39) [5] (difference in log means = −0.02; 95% CI −0.1 to 0.1, *p* = 0.57). When stratified into 10‐year age groups, there was no evidence of any differences in GM in any adult age group compared to previous measures.

#### 4.1.2 Children

Only children with a venous BLL measurement were included in the analysis; children with a capillary screening result below the threshold were excluded, which may result in overestimation of BLLs in younger age groups. Young children aged 0–4 years (*n* = 26) had a GM BLL of 1.82 µg/dL (95% CI 1.36–2.44). There have been no recent studies measuring BLLs in children under the age of 5 years in NZ, so a comparison is not possible for this age group. International data has reported BLLs higher in young children aged up to 5 years, compared to those aged 6–11 years [20].

When stratified by age group, children aged 5–9 years (*n* = 53) had a GM BLL of 1.06 (95% CI 0.92–1.22), which is not significantly different from previous measures in NZ children in this age group [5] (*p* = 0.19). The GM of children aged 10–17 years (*n* = 73) was 0.97 (0.86–1.08), which is also not significantly higher than prior estimates from all of NZ [5] (*p* = 0.07).

Multivariable analysis showed that higher BLLs in children were associated with older homes (built pre‐1945), the presence of peeling paint or recent renovations. In adults, higher BLLs were associated with increasing age and high‐risk occupations. Although BLLs in younger children were somewhat higher than previous estimates, this could have been influenced by factors such as the age of the housing stock [21], and seasonality. This study was undertaken over a short period in summer in a rural area—it is possible that children would spend more time outdoors compared with other studies and potentially have been exposed to more environmental lead from sources such as peeling paint and soil [22, 23].

**FIGURE 3:**
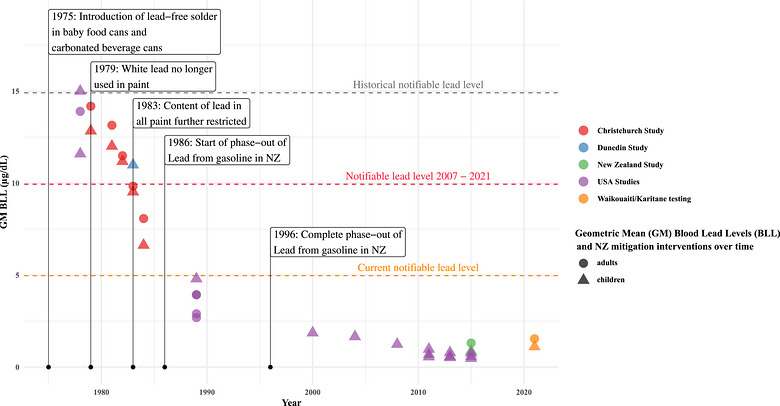
Geometric mean (GM) blood lead levels (BLL) and New Zealand mitigation interventions over time. NZ, New Zealand.

The BLLs measured in East Otago adults and children in 2021 were much lower than levels recorded in NZ in the 1980–1990s (Figure 3), reflecting the effective public health actions taken to remove lead from the environment.

### 4.2 International Comparisons

These BLLs measured in East Otago for adults and children (1.48 µg/dL (1.43–1.53)) are higher than those recently recorded in similar high‐income countries, such as the United States in 2017/2018 (0.75 µg/dL) (0.72–0.78) and Canada in 2019 (0.81 µg/dL) (0.77–0.85 µg/dL) [20, 24]. This is likely due to the lag in removing lead from petrol, occupational exposure, the continued presence of lead‐based paint on older houses, lead‐contaminated soil and national plumbing standards that allow the use of small amounts of lead in the manufacture of plumbing fixtures. The East Otago findings are consistent with previous work undertaken in NZ in 2014–2016, which also identified that BLLs in NZ children and adults were higher than international comparisons [5, 25].

### 4.3 Strengths and Limitations

The high uptake of BLL testing means that the findings are likely to be representative, and any effect of selection bias will have been minimal. There was slight undersampling of males compared with females, which may have biased results. Because younger children were more likely to be offered capillary testing, venepuncture sampling is biased towards older children, so there are low numbers of children aged 0–5 years in the final analysis. Although capillary testing is subject to contamination, it was used for screening purposes only, and all analyses were based on venous BLLs. Because children with elevated screening levels all received follow‐up venepuncture, this may have selected children with higher BLLs to be differentially included in the final analysis, resulting in an upwards bias of the GM in young children.

The findings of this study have limited generalisability. The population had a high proportion of older adults residing in one semi‐rural area in southern NZ. The rurality may have influenced the likelihood of lead exposure from soil or from occupational sources and from older housing.

There was no evidence that the intermittent elevated lead levels detected in the drinking water were associated with consistently elevated BLLs in the exposed population. The small number of people with BLLs above the notifiable threshold suggests that routine screening is unlikely to be of benefit. However, this study indicates that BLLs in NZ are still substantially higher than in other comparable high‐income countries—likely due to lead exposure from known sources such as old paint and tapware. These findings contributed to a change in the tapware legislation in NZ that will come into effect in 2025; the proportion of lead permitted in tapware will be reduced to 0.25% by weight [26]. Although much progress has been made in recent times to reduce environmental lead, it remains important to increase public awareness of possible exposure, such as contact with flaking lead‐based paint during home renovations, and to flush taps before consuming water.

## Author Contributions

**Melyssa Roy**: investigation, writing – original draft, writing – review and editing. **Michael Butchard**: conceptualisation, investigation, methodology, supervision, project administration, writing – review and editing. **Susan Jack**: investigation, conceptualisation, writing – review and editing, project administration, supervision, methodology. **Callum Thirkell**: investigation, conceptualisation, writing – review and editing, methodology, formal analysis, data curation. **Giles Graham**: conceptualisation, investigation, validation, methodology, visualisation, writing – review and editing, formal analysis, data curation. **Andrew Anglemyer**: conceptualisation, investigation, writing – review and editing, methodology, validation, data curation, visualisation, formal analysis.

## Funding

The New Zealand Ministry of Health funded the health response as part of the public health service.

## Disclosure

The study design, data collection, analysis, interpretation, writing and submission of the report has been undertaken independently by the authors.

## Ethics Statement

This work uses data collected as part of a public health response, so no formal ethical approval was sought. The study was conducted in accordance with recognised ethical standards for such investigations.

## Consent

All participants have given written consent for de‐identified reporting of results.

## Conflicts of Interest

The authors declare no conflicts of interest.

## Data Availability

The data collected for this study were part of a public health investigation and are therefore cannot be publicly shared.
